# Validity of the Brazilian online version of the Sexual Desire Inventory 2

**DOI:** 10.1590/1806-9282.20240362

**Published:** 2024-07-19

**Authors:** Denisse Cartagena-Ramos, Miguel Fuentealba-Torres, Luiz Henrique Arroyo, Daniella Talita dos Santos, Flávio Rebustini, Lúcia Alves Silva Lara, Ricardo Alexandre Arcêncio, Lucila Castanheira Nascimento

**Affiliations:** 1 Andrés Bello University, Faculty of Nursing – Santiago, Chile.; 2 Universidad de los Andes, Faculty of Nursing and Midwifery – Santiago, Chile.; 3 Secretariat of Health Surveillance, Ministry of Health – Brasília (DF), Brazil.; 4 School of Public Health of Paraná, Department of Health of Paraná – Curitiba (PR), Brazil.; 5 Universidade de São Paulo, School of Arts, Sciences and Humanities – São Paulo (SP), Brazil.; 6 Universidade de São Paulo, Ribeirão Preto Medical School – Ribeirão Preto (SP), Brazil.; 7 Universidade de São Paulo, Ribeirão Preto College of Nursing – Ribeirão Preto (SP), Brazil.

**Keywords:** Libido, Reproducibility of results, Psychological tests, Psychometric, Sexual health

## Abstract

The Sexual Desire Inventory 2 is a self-report instrument for assessing sexual desire in men and women. In Brazil, there is no validated sexual desire self-report for the adult population.

**Objective:**

The aim of this study was to determine the evidence of validity for the content and construct of the Brazilian online version of the Sexual Desire Inventory 2.

**Methods:**

This was a cross-sectional study with Brazilian men and women. The sample size was calculated using the criterion of more than 20 participants per item. The invitation to participate in the study was conducted online by the platform Survey Monkey^®^. The Sexual Desire Inventory 2 was evaluated for content, construct, reliability, and invariance.

**Results:**

A total of 818 female and male adults participated in the study. The two-dimensional factorial solution represented 71% of the total variance explained by the model, and the factorial loads of the model were ≥0.40; commonalities presented values ≥0.23. Reliability was measured by the coefficients of Cronbach's alpha with a total score of 0.87, McDonald's of 0.87, Omega, and greatest lower bound with a total score of 0.95. The metric invariance was tested for the sex variables ΔCFI (comparative fit index) and ΔRMSEA (root mean square error of approximation) with a total score of 0.01.

**Conclusion:**

The analyses indicate evidence of robust validity in the Brazilian online version of the Sexual Desire Inventory 2.

## INTRODUCTION

The World Association of Sexual Health recently adopted sexual pleasure, defined as "the physical and/or psychological satisfaction and enjoyment derived from shared or solitary erotic experiences, including thoughts, fantasies, dreams, emotions, and feelings," as the cornerstone of sexual health^1^.

In Brazil, two studies showed that the most relevant problem is low sexual desire^2,3^; because of the Hypoactive Sexual Desire Disorder has been associated with biological and psychological causes^4^, validated instruments of measurement are essential to adequately assess sexual desire in the population^5^ by determining the prevalence of estimates and showing the evidence of the problem. However, there are no validated online instruments to measure sexual desire or the construct of sexual desire in Brazil^6^. In addition, the measurement of the evaluation of sexual desire through the use of multi-domain instruments of sexual function is feasible. However, it may not be adequate to evaluate the construct of sexual desire^7^ because it can potentially compromise some of its psychometric properties^8,9^. The Sexual Desire Inventory 2 (SDI-2)^10^ is a measuring instrument that has been adapted to other cultures^11-13^ and has now been culturally adapted and validated for the Brazilian population. Therefore, the present study aimed to demonstrate evidence of the validity of the Brazilian online version of the SDI-2.

## METHODS

### Study design

This was a cross-sectional study conducted between May and October 2018, with Brazilian men and women, to determine the evidence of validity for the content and construct of the Brazilian online version of the SDI-2.

### Participants and procedures

Participants were selected based on the following inclusion criteria: women and men over 18 years of age, literate, and capable of understanding the content of the SDI-2. The sample size was calculated using the criterion of more than 20 participants per item in the SDI-2^8^.

The invitation to participate in the study was conducted online by sending a URL (uniform resource locator) link made available through the social networks Facebook^®^ and Twitter^®^ and by e-mail invitations. The link directed users to the invitation to participate in the study and, subsequently, to the platform Survey Monkey, where participants had access to the Informed Consent Terms (TCLE).

### Exploratory factor analysis

The adequacy of the correlation matrix was evaluated through Bartlett's statistic and Kaiser-Meyer-Olkin (KMO) tests and analyzed using the polychoric correlation and considering the amplitude of the scale from 0 to 8^8^.

For the dimensionality testing, parallel analysis was applied through the optimal implementation of parallel analysis. In addition, the UNICo (one-dimensional congruence) >0.95; the ECV (explained common variance) >0.85; or the MIREAL (mean of item residual absolute loading) <0.30^8^ was used to confirm if the model was unidimensional or multidimensional.

The robust unweighted least squares was used for data extraction, associated with a bootstrap (n=5,000) and the direct oblimin rotation. The two-dimensional model was adopted as the initial model and as the original instrument. Factorial solutions were evaluated by factorial saturation >0.40, with total explained variance >60%, and commonalities >0.40^8^.

Pratt's importance measures^14^ were used as a way of complementing the factorial solution. This method helps to solve three difficulties of interpretation that arise in oblique models. First, it integrates the information between the standard and structure coefficients. Second, it restores horizontal and vertical addition properties while allowing factors to be oblique. Third, it solves, in part, the traditional problem of rules to evaluate the meaning of the relationship between the observed variable and the factor^8^.

The confirmatory factor analysis (CFA) was evaluated by the factorial model index adjustments, the root mean square error of approximation (RMSEA) ≤0.006, the non-normed fit index (NNFI; Tucker & Lewis) >0.95, the comparative fit index (CFI) >0.95, the goodness-of-fit index (GFI) >0.95, and the adjusted goodness-of-fit index (AGFI) >0.95^15^.

### Reliability, quality, and replicability of the factorial solution

Reliability was evaluated by the coefficients of Cronbach's alpha, the greatest lower bound (GLB), and McDonald's Omega.

The quality of the factorial solution and replicability of the model were tested by the generalized H (GH) index, and the quality and effectiveness of estimates of factors' scores were calculated by the factor determinacy index (FDI) and the ORION marginal reliability^8^.

### Invariance

The metric invariance was tested with the ΔCFI and ΔRMSEA between a sample of men and women. The difference between models should not be greater than 0.01 for ΔCFI and 0.015 for ΔRMSEA^16^.

Study approval by the University Institutional Review Board was obtained prior to commencing the study (CAAE number 79325517.2.0000.5393). Additionally, all participants signed an online free and informed consent form according to Resolution 466/12 of the Brazilian National Council of Health.

### Measures

#### Sociodemographic characteristics

A structured sociodemographic and clinical questionnaire comprising 12 questions and including personal data such as date of birth, country of residence, sex, marital status, education, occupation, race, history of chronic illness, religion, relationship length, sexual preference, and frequency of sexual activity was used.

#### Sexual Desire Inventory 2

The Brazilian version of the Sexual Desire Inventory 2 was applied to determine evidence of validity. The cultural adaptation of the instrument, which preceded the present validation study, has been previously reported in detail. The Brazilian version of the Sexual Desire Inventory 2 includes 14 items: 4 of them with scores ranging from 0 to 7 and related to the frequency of desire, and the remaining 10 items are answered on a scale with scores ranging from 0 to 8. The scores from items 1 through 8 are added to obtain the sexual desire score in a relationship, while scores from items 9 through 11 are added to obtain the solitary sexual desire score. SDI-2 scores range from 0 to 112^10^.

### Data analysis

The statistical analyses were performed using the FACTOR software version 10.8.04 with a statistical power of 95% and a significance index of 0.05, and the IBM SPSS AMOS software version 22.0 with a statistical power of 95% and a significance index of 0.05. The descriptive statistical analyses of the sociodemographic variables were performed, and the minimum and maximum frequencies and percentages were calculated. Measurements of central tendency and dispersion were calculated for the variable of age.

## RESULTS

A total of 960 participants were recruited, of whom 818 agreed to participate. Out of these, 142 participants were excluded due to the incomplete filling of collection instruments, and the final sample comprised 818 subjects. Of note, 65.8% (n=538) were women and 34.2% (n=280) were men. Table 1 shows the sociodemographic characteristics of the study participants.

**Table 1:** Descriptive characteristics of the respondents (n=818).

Characteristics		min–max	n (%)
Chronic disease			
	No		672 (82.2)
	Yes		146 (17.8)
Religion (active participation)			
	No		521 (63.7)
	Yes		297 (36.3)
Relationship			
	No		301 (36.8)
	Yes		517 (63.2)
Sexual preference			
	By women		187 (22.9)
	By men		487 (59.5)
	For men and women		130 (15.9)
	Rather not answer		14 (1.7)
Sexual activity			
	Two or three times a month		147 (18.0)
	Twice a week		136 (16.6)
	More than once a day		13 (1.6)
	Not once		111 (13.6)
	Three or four times a week		119 (14.5)
	Once a month		130 (15.9)
	Once a day		24 (2.9)
	Once a week		138 (16.9)

### Construct validity

The suitability of the sample pointed to a KMO=0.85 and Bartlett's statistics value of 74.7 (p<0.010), indicating the good factorability of the data. The analysis of dimensionality performed by the robust parallel analysis indicated the existence of two dimensions. The complementary indicators for dimensionality also indicated a multidimensional model with UNICo=0.873; ECV=0.675, and MIREAL=0.383.

The two-dimensional factorial solution represented 71% of the total variance explained by the two-dimensional model. The configuration was defined as Factor 1 (responsive sexual desire interpreted as sexual desire in the relationship) retaining items 1, 2, 3, 4, 5, 6, 7, 8, and 9, and Factor 2 (related to spontaneous sexual desire interpreted as solitary sexual desire) retaining items 10, 11, 12, and 13. Table 2 presents the values of factorial loads, commonalities, and Pratt's measures. Table 3 presents the adjustment index values observed in the one- and two-factor models of the CFA.

**Table 2:** Standardized factor loadings, communalities (h2), and confirmed factorial solutions from the exploratory factorial analysis.

Item number of the Sexual Desire Inventory 2 (SDI-2)	Número do item do Inventário de Desejo Sexual 2 (IDS-2)	Factor loading		Communalities	Pratt's measure	
		F1	F2	h2	F1	F2
1	1	0.64	0.04	0.43	0.42	
2	2	0.62	0.12	0.45	0.41	
3	3	0.81	-0.05	0.64	0.64	
4	4	0.44	0.14	0.25	0.21	
5	5	0.40	0.16	0.23	0.18	
6	6	0.57	-0.12	0.30	0.30	
7	7	0.86	0.01	0.73	0.73	
8	8	0.65	0.03	0.43	0.43	
9	9	0.80	-0.00	0.64	0.64	
10	10	0.12	0.74	0.63		0.58
11	11	-0.00	0.91	0.83		0.83
12	12	-0.06	0.92	0.81		0.81
13	13	0.01	0.88	0.79		0.79

F1: dyadic sexual desire; F2: solitary sexual desire; h2: communalities; Pratt's importance measures; p<0.05.

**Table 3:** Summary of goodness-of-fit statistics for Sexual Desire Inventory 2.

Model CFA	X^2^	df	X^2^/df		RSMEA	NNFI	CFI	GFI	AGFI
Two factors	339.133	53	6.39		0.121	0.91	0.939	0.979	0.968
**Metric invariance across sex for SDI-2**	**Model**	**n**	**X^2^**	**Df**	**X^2^/df**	**CFI**	**RMSEA**	**ΔCFI**	**ΔRMSEA**
Female	Two factor	538	217.078	53	4.09	0.941	0.123	0.001	0.013
Male	Two factor	280	145.469	53	2.74	0.94	0.11		

CFA: confirmatory factor analysis; RMSEA: root mean square error of approximation; NNFI: non-normed fit index; CFI: comparative fit index; GFI: goodness-of-fit index; AGFI: adjusted goodness-of-fit index.

The factorial loads of the model were ≥0.40, and commonalities presented values ≥0.23. The technique of Pratt's measures reaffirmed the alignment of items in two factors, corroborating the solution proposed in the factorial analysis.

### Reliability, quality, and replicability of the factorial solution

Reliability was evaluated by the values of the Cronbach's alpha coefficient for the instrument, with a total score of 0.87; for the subscale of desire in a relationship, 0.84; and for the subscale of solitary desire, 0.91. The McDonald's Omega coefficient value was 0.87, and the GLB coefficient value was 0.95.

The stability of the Brazilian version of the SDI-2 was evaluated through the GH index, with a value of 0.90 for the subscale of solitary desire and a value of 0.93 for the subscale of sexual desire in a relationship. The quality and effectiveness of estimates were evaluated through the FDI, which indicated the values of 0.95 and 0.96, and through the ORION marginal reliability, which indicated the values of 0.90 and 0.93 for the first and second factors, respectively. All indicators were above the stipulated minimum limits.

#### Invariance

The metric invariance (Table 3) showed stability between the models for the female and male genders. The ΔCFI and ΔRMSEA resulted in 0.01, that is, within limits established in the literature.

## DISCUSSION

The present study aimed to demonstrate evidence of the validity of the online version of the SDI-2 instrument. Furthermore, the increase in the validation of measurement instruments has impacted new proposals for cultural adaptation and/or validations of online versions, which brings multiple advantages^9,17^.

One study showed that web-based data collection does not statistically increase or decrease the consistency of responses, nor does it compromise the integrity of the test, and it is a suitable alternative to more traditional methods^18^.

Corroborating the results found in the present study, some validation studies have demonstrated adequate results using different psychometric techniques^19-21^. The choice of techniques applied in this study aimed at increasing the accuracy and consistency of analyses^8,16,22,23^.

## CONCLUSION

The online version of the SDI-2 is a self-report that presents satisfactory, and at first, stable, construct validity evidence with a final model composed of 14 items and divided into two dimensions.

Future studies using the Brazilian online version of the SDI-2 may be essential to estimate the prevalence of sexual desire disorder in men and women and to identify effective interventions that promote sexual health and well-being in the Brazilian adult population.
